# Supplementary material for: Trans Species RNA Activity: Sperm RNA of the Father of an Autistic Child Programs Glial Cells and Behavioral Disorders in Mice
Source: Biomolecules. 2024 Feb 7;14(2):201. doi: 10.3390/biom14020201 (PMC10886764; doi:10.3390/biom14020201)
Supplement: Supplementary file 1 [file biomolecules-14-00201-s001.zip › biomolecules-2764435-supplementary.pptx]

## Slide 1
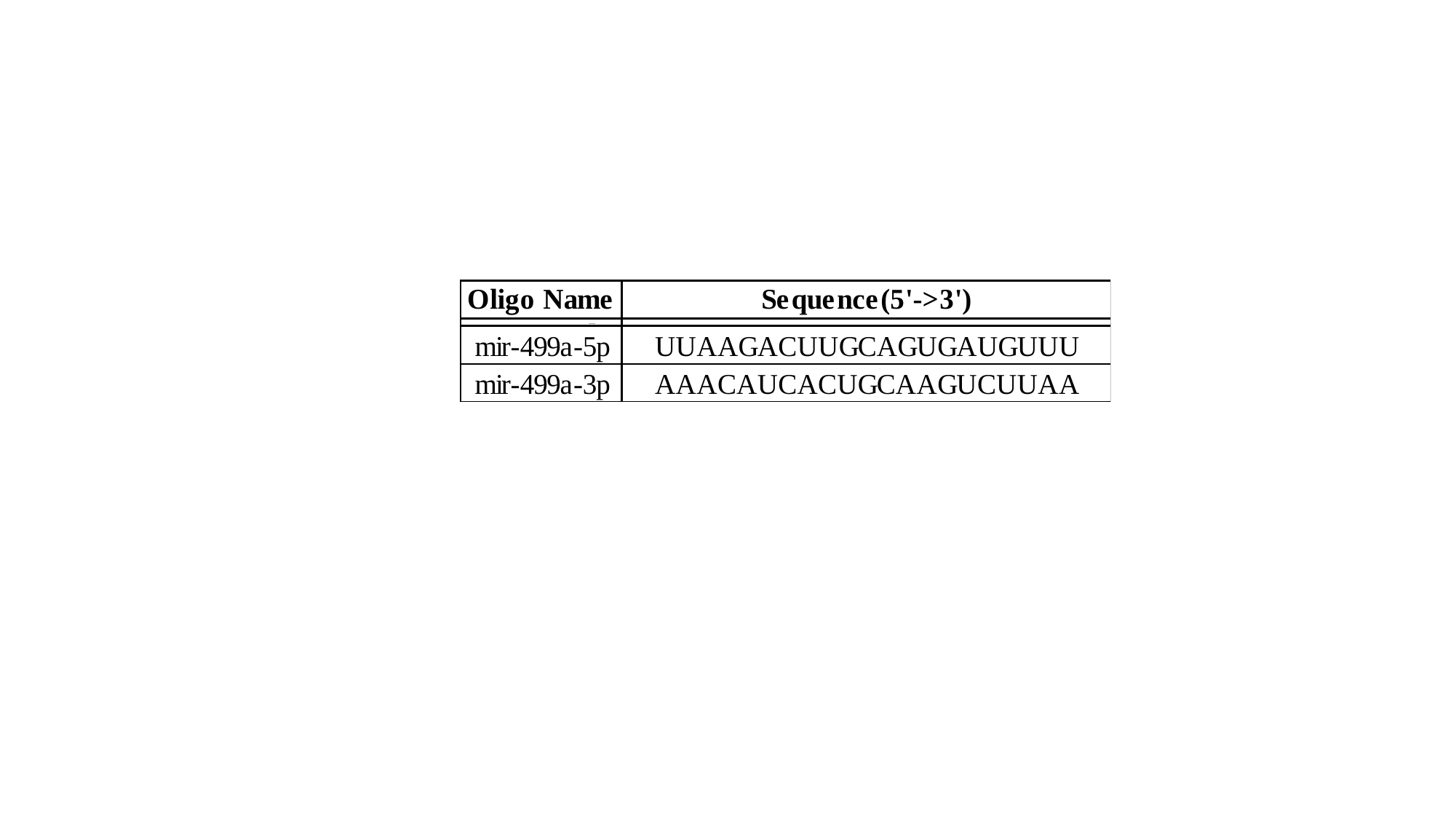

## Slide 2
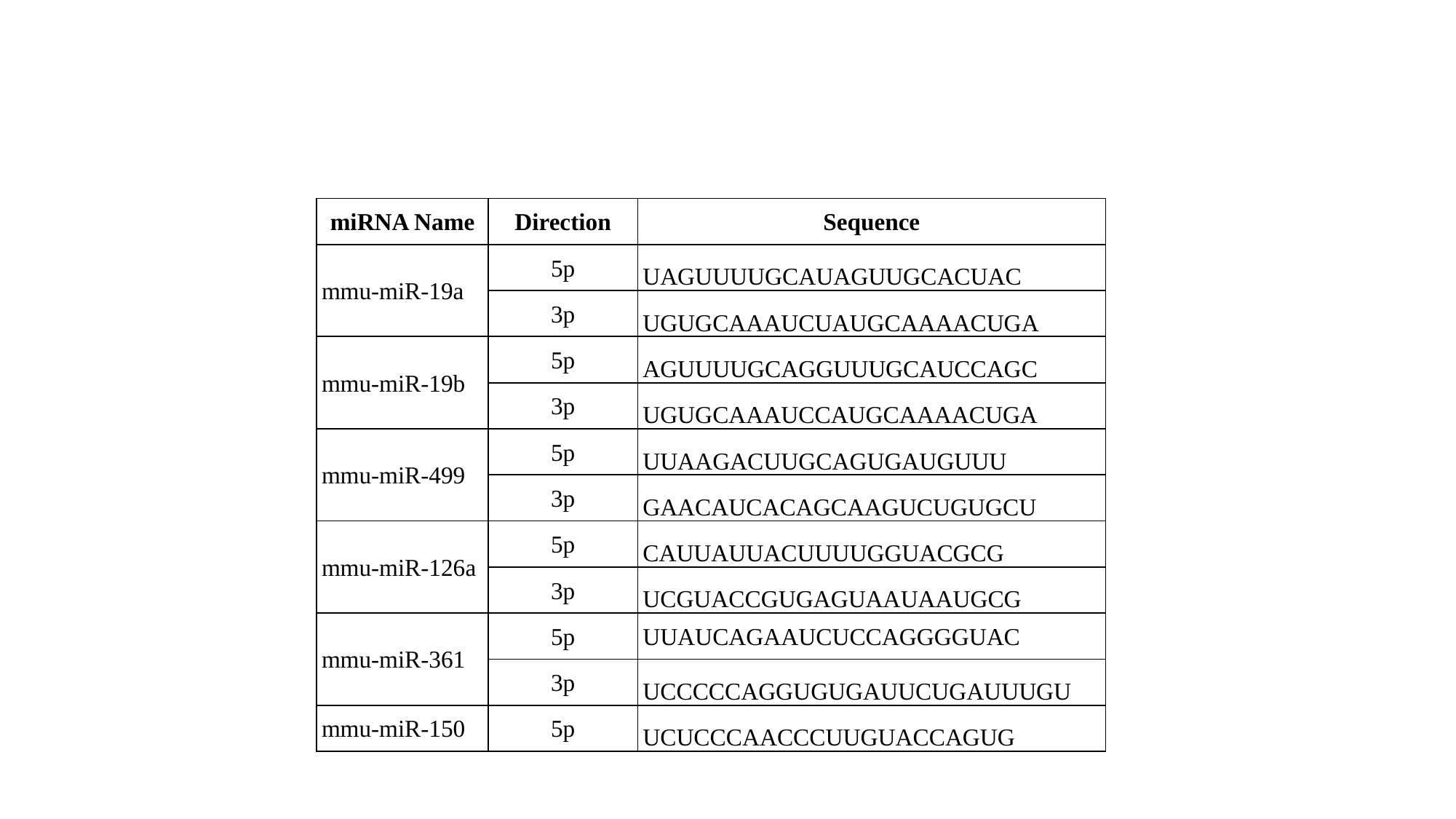

| miRNA Name | Direction | Sequence |
| --- | --- | --- |
| mmu-miR-19a | 5p | UAGUUUUGCAUAGUUGCACUAC |
| | 3p | UGUGCAAAUCUAUGCAAAACUGA |
| mmu-miR-19b | 5p | AGUUUUGCAGGUUUGCAUCCAGC |
| | 3p | UGUGCAAAUCCAUGCAAAACUGA |
| mmu-miR-499 | 5p | UUAAGACUUGCAGUGAUGUUU |
| | 3p | GAACAUCACAGCAAGUCUGUGCU |
| mmu-miR-126a | 5p | CAUUAUUACUUUUGGUACGCG |
| | 3p | UCGUACCGUGAGUAAUAAUGCG |
| mmu-miR-361 | 5p | UUAUCAGAAUCUCCAGGGGUAC |
| | 3p | UCCCCCAGGUGUGAUUCUGAUUUGU |
| mmu-miR-150 | 5p | UCUCCCAACCCUUGUACCAGUG |

## Slide 3
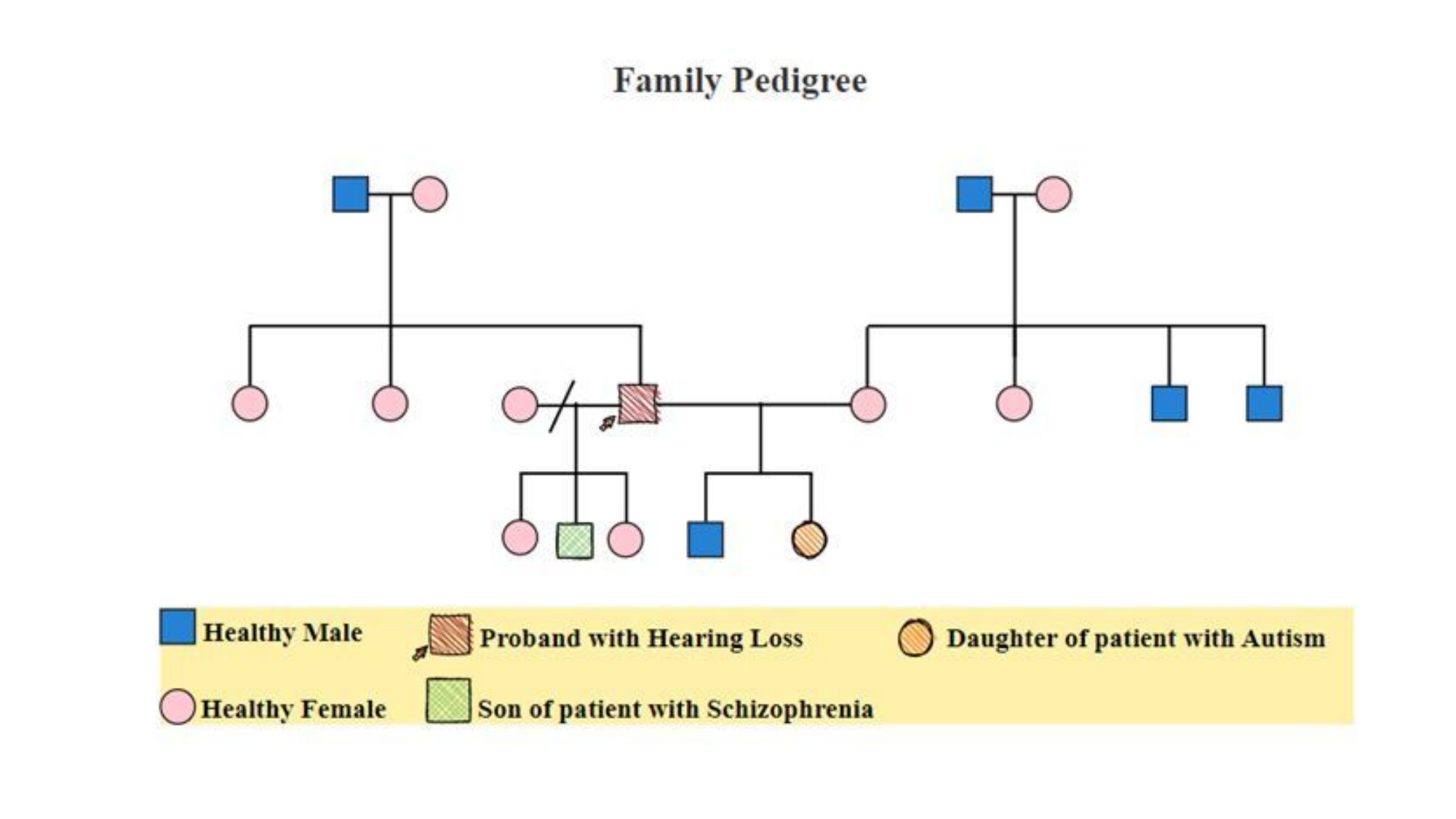

## Slide 4
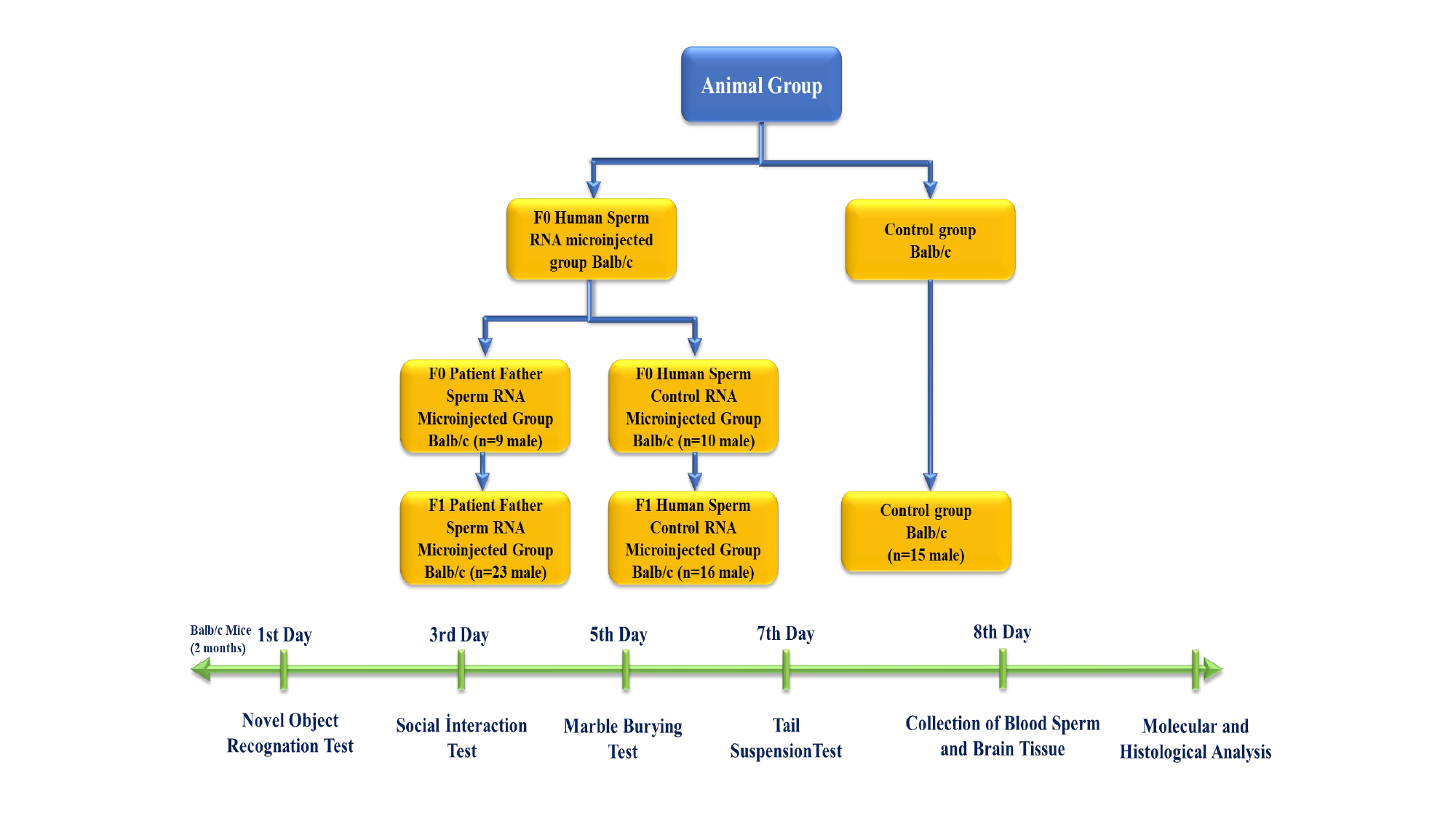

## Slide 5
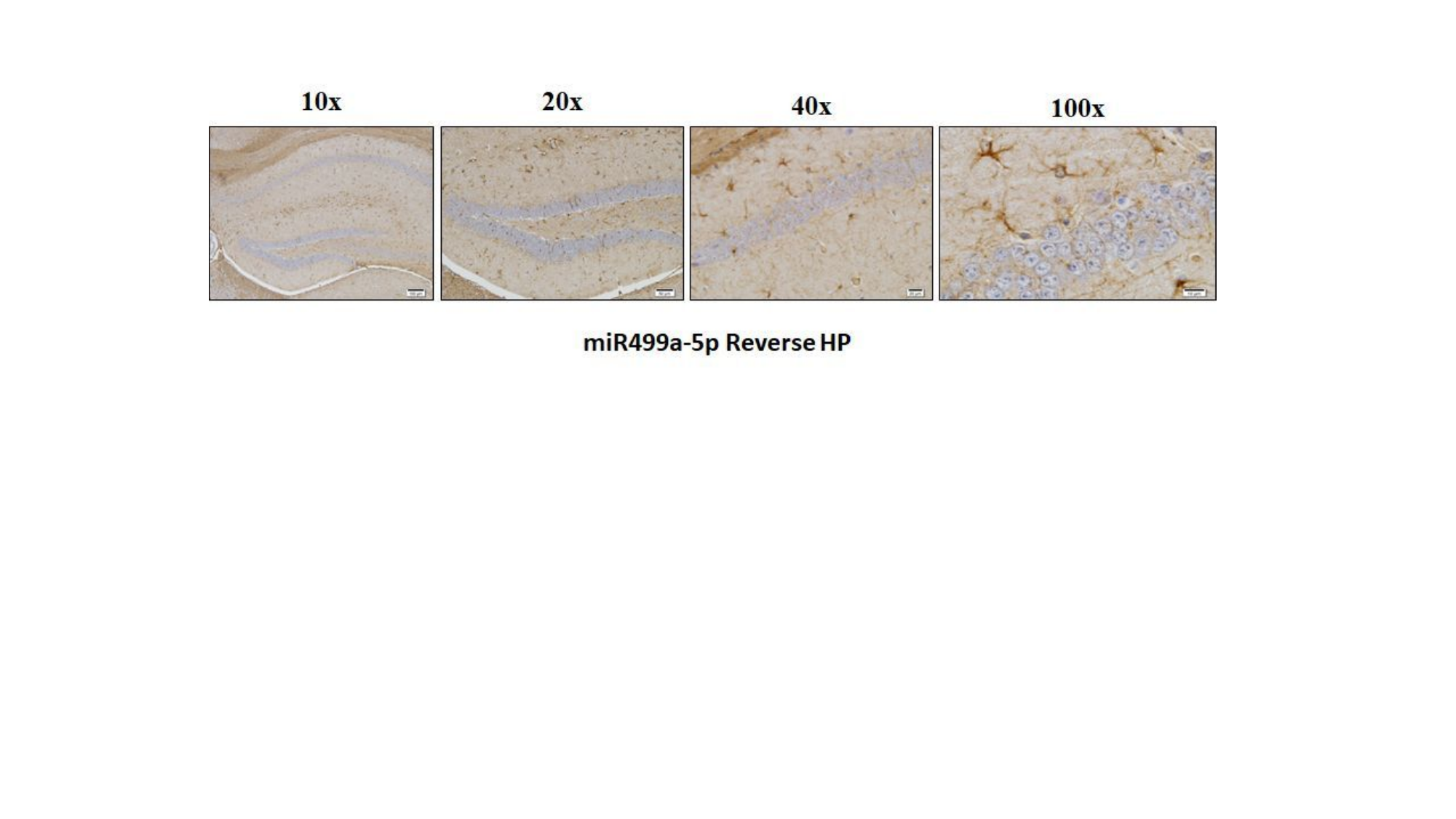

## Slide 6
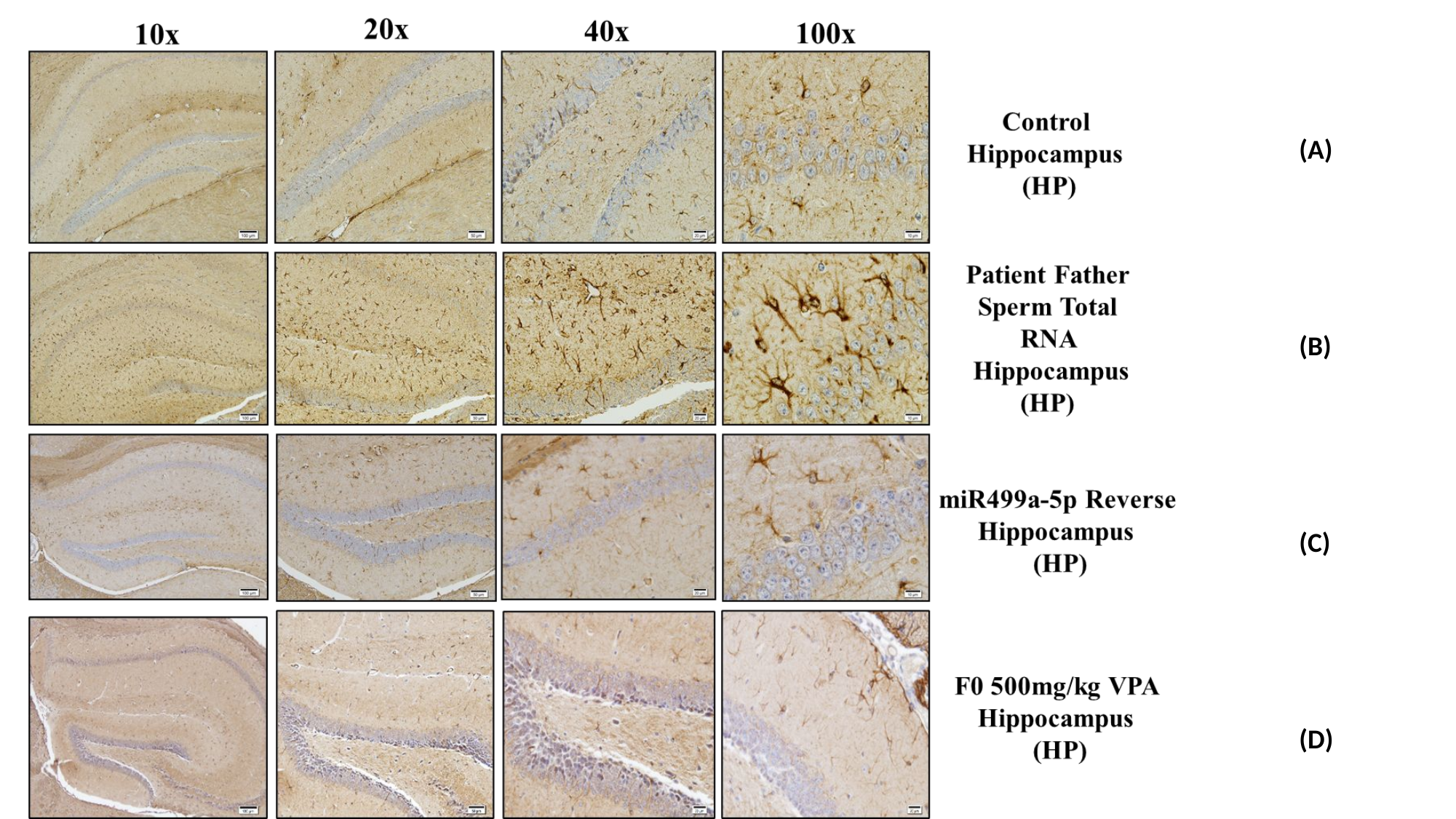

(A)
(B)
(C)
(D)

## Slide 7
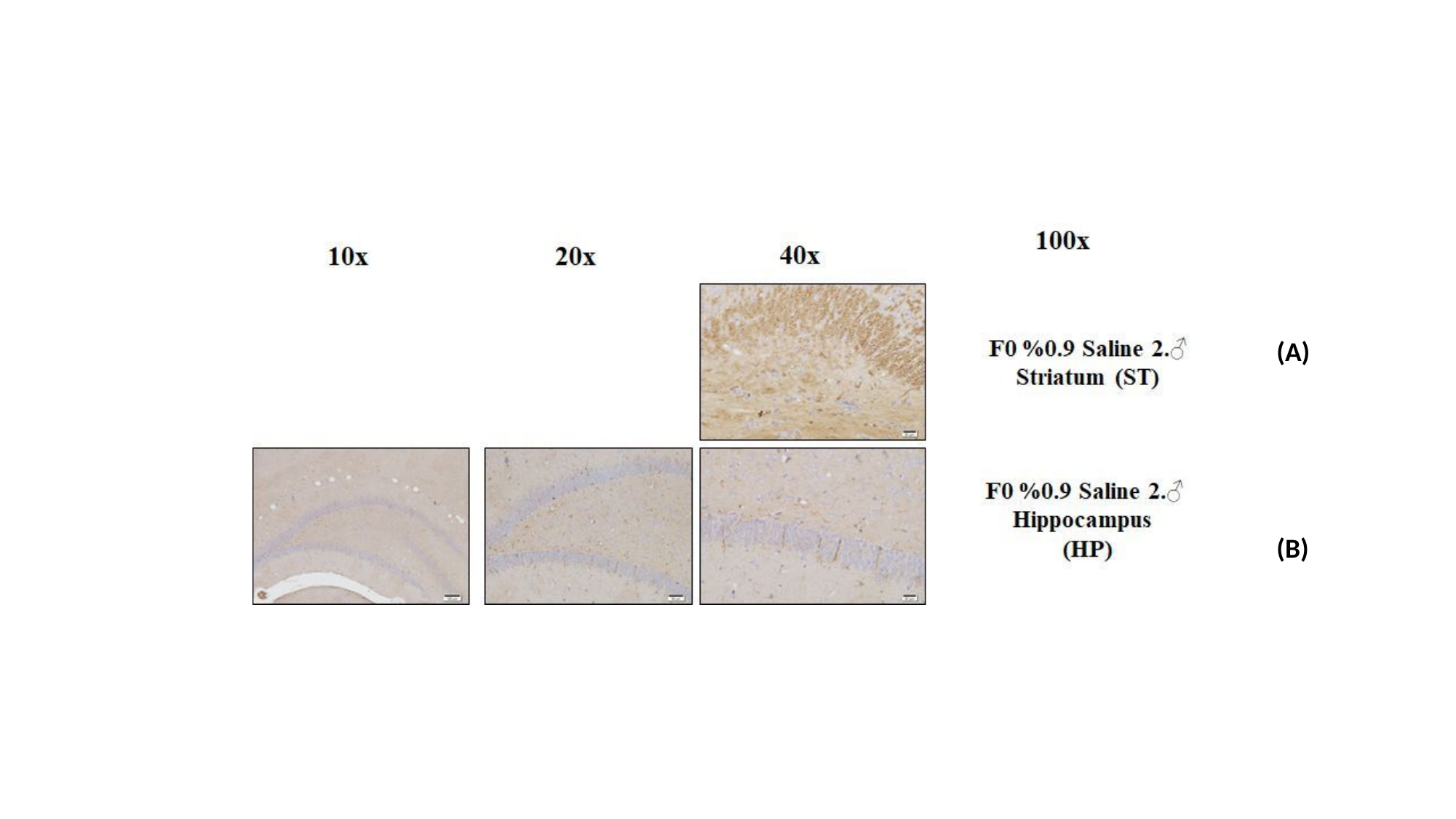

(A)
(B)

## Slide 8
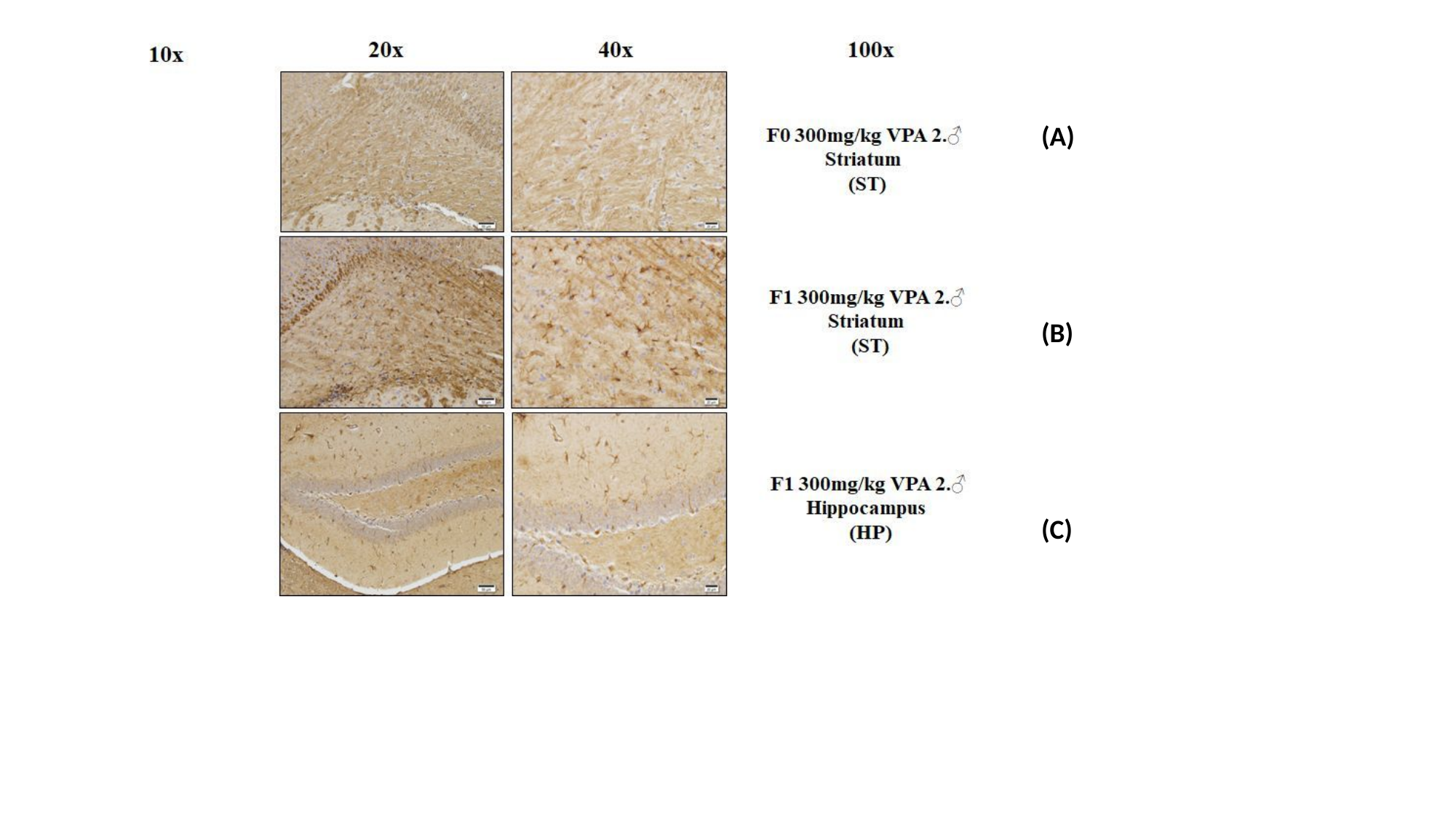

(A)
(B)
(C)

## Slide 9
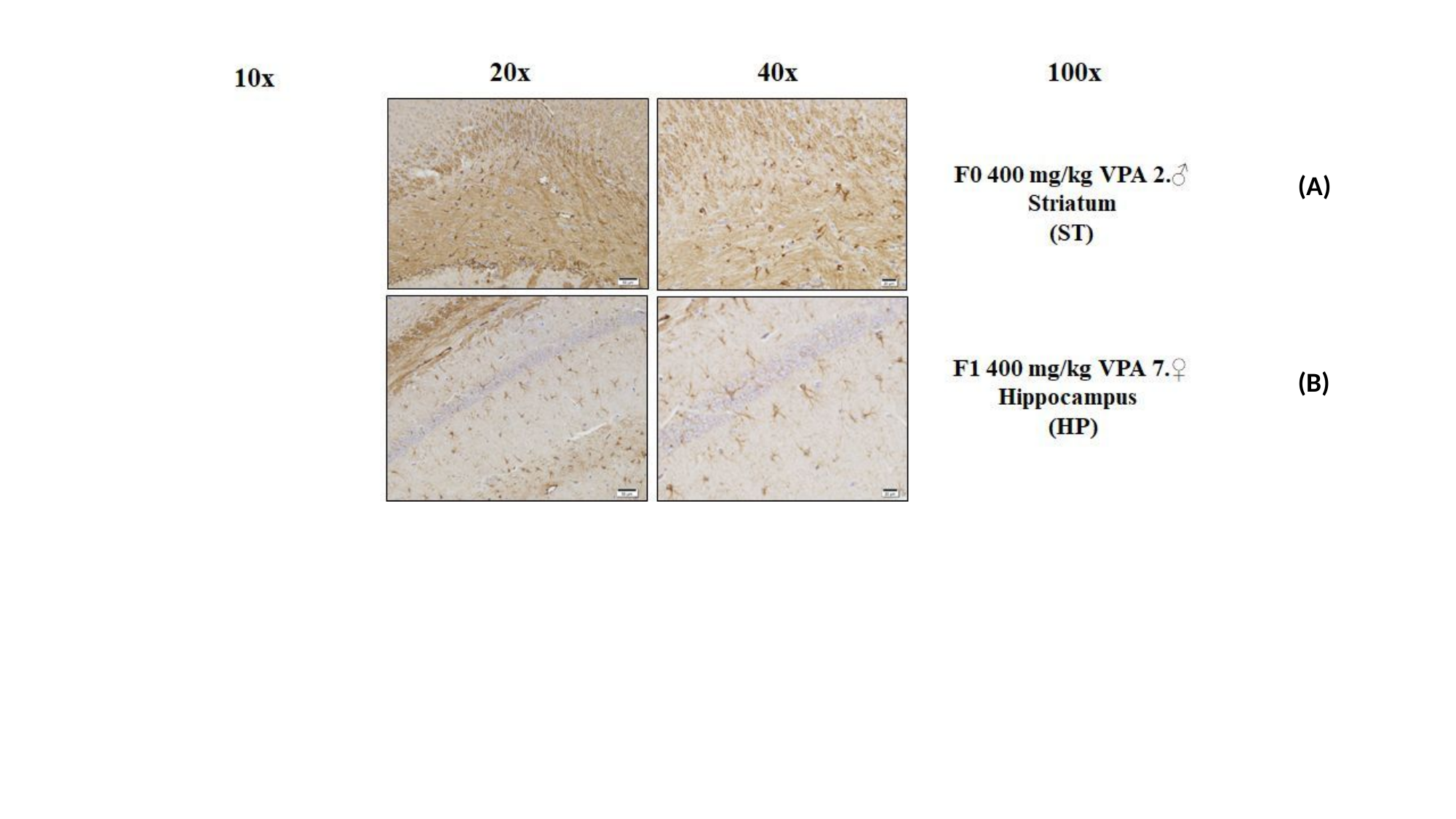

(A)
(B)

## Slide 10
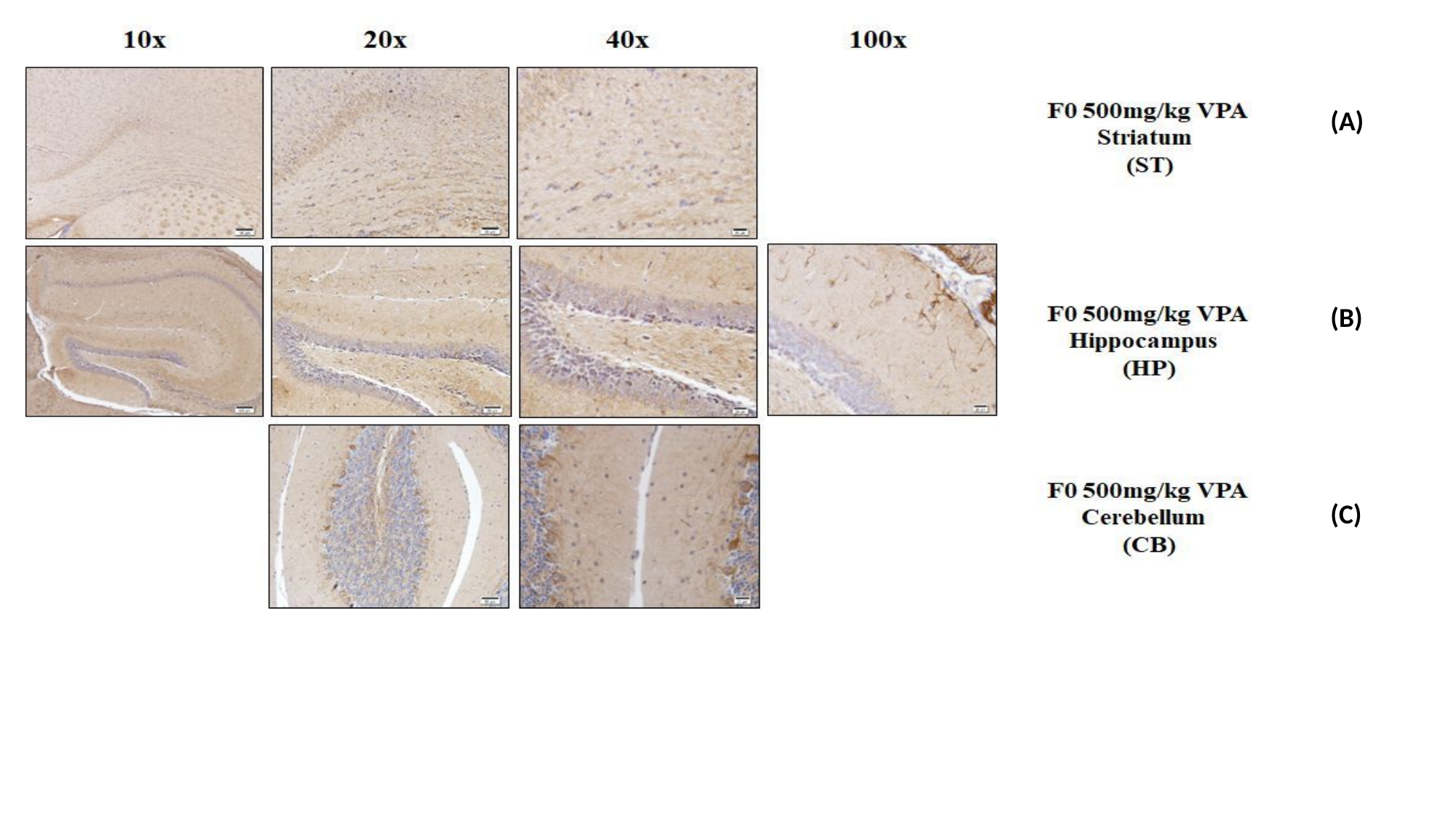

(A)
(B)
(C)

## Slide 11
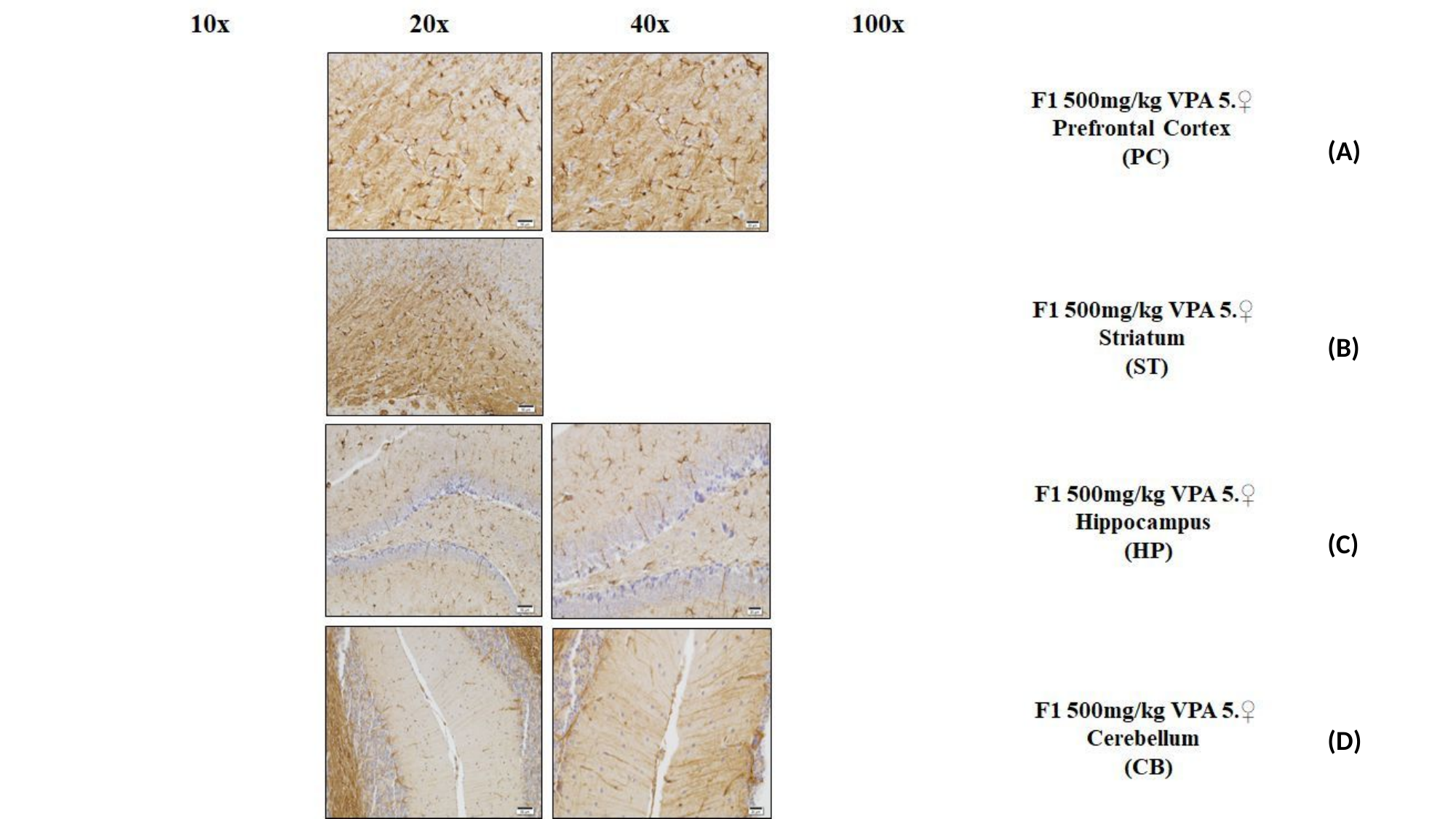

(A)
(B)
(C)
(D)

## Slide 12
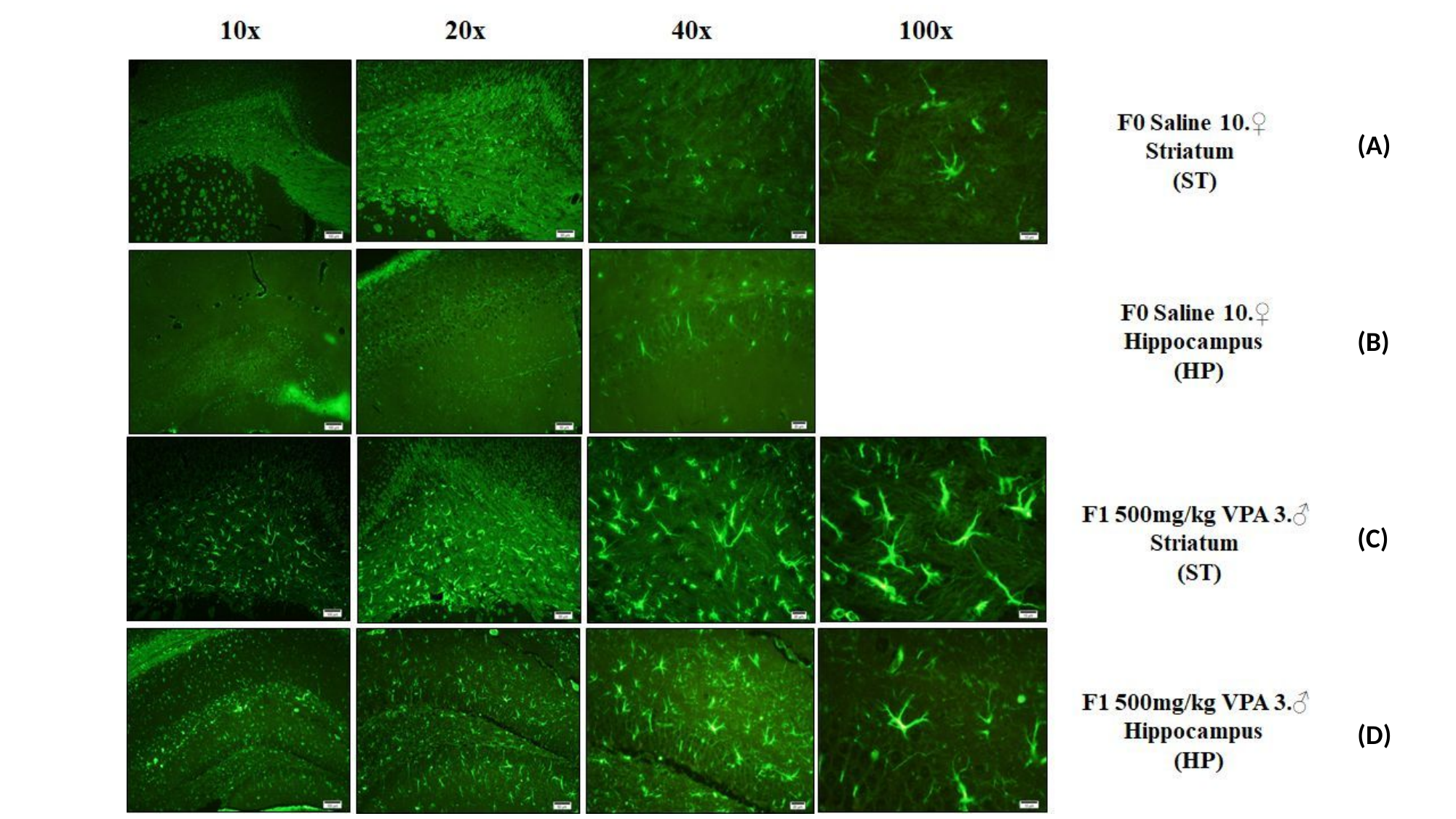

(A)
(B)
(C)
(D)

## Slide 13
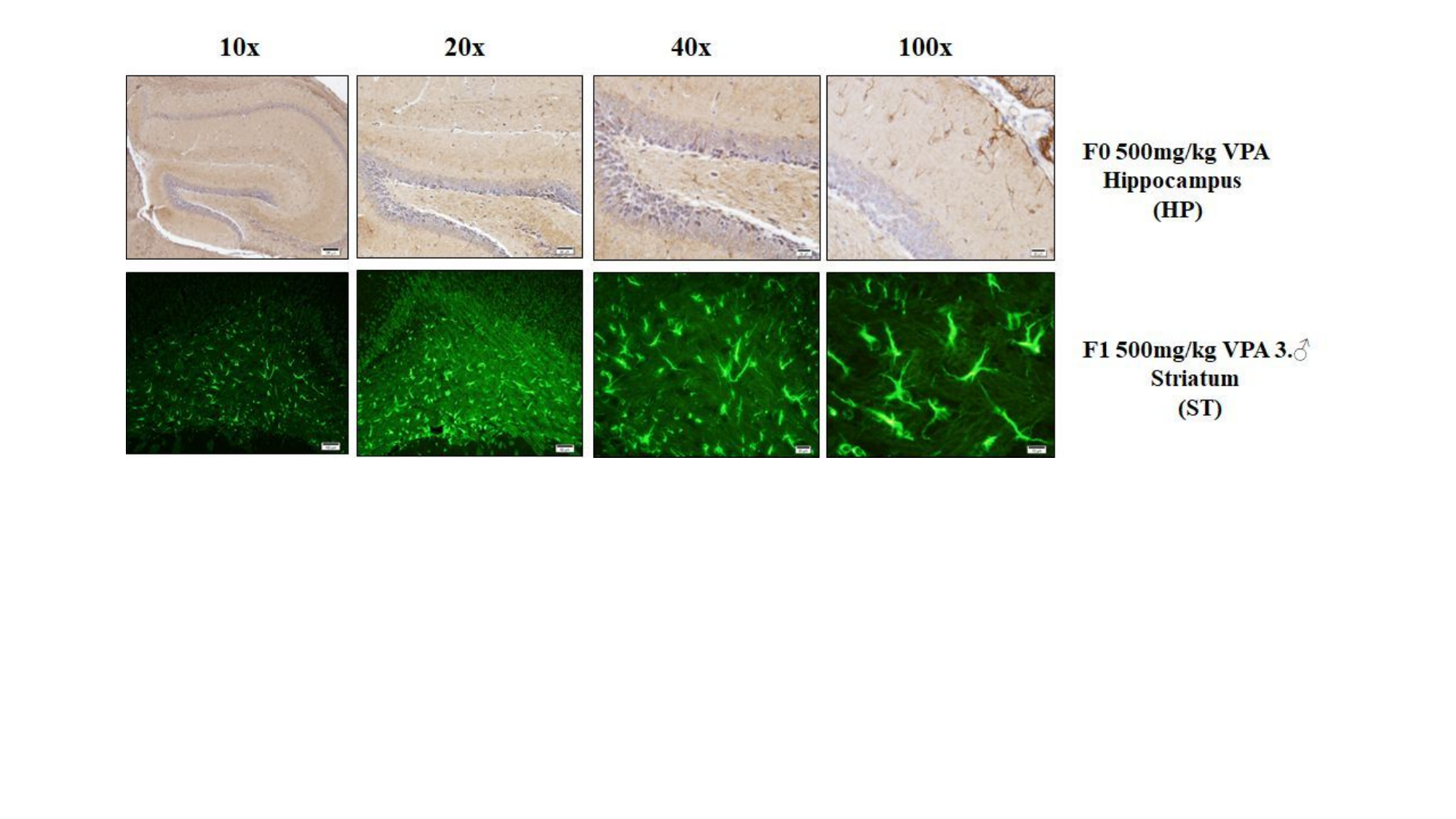

## Slide 14
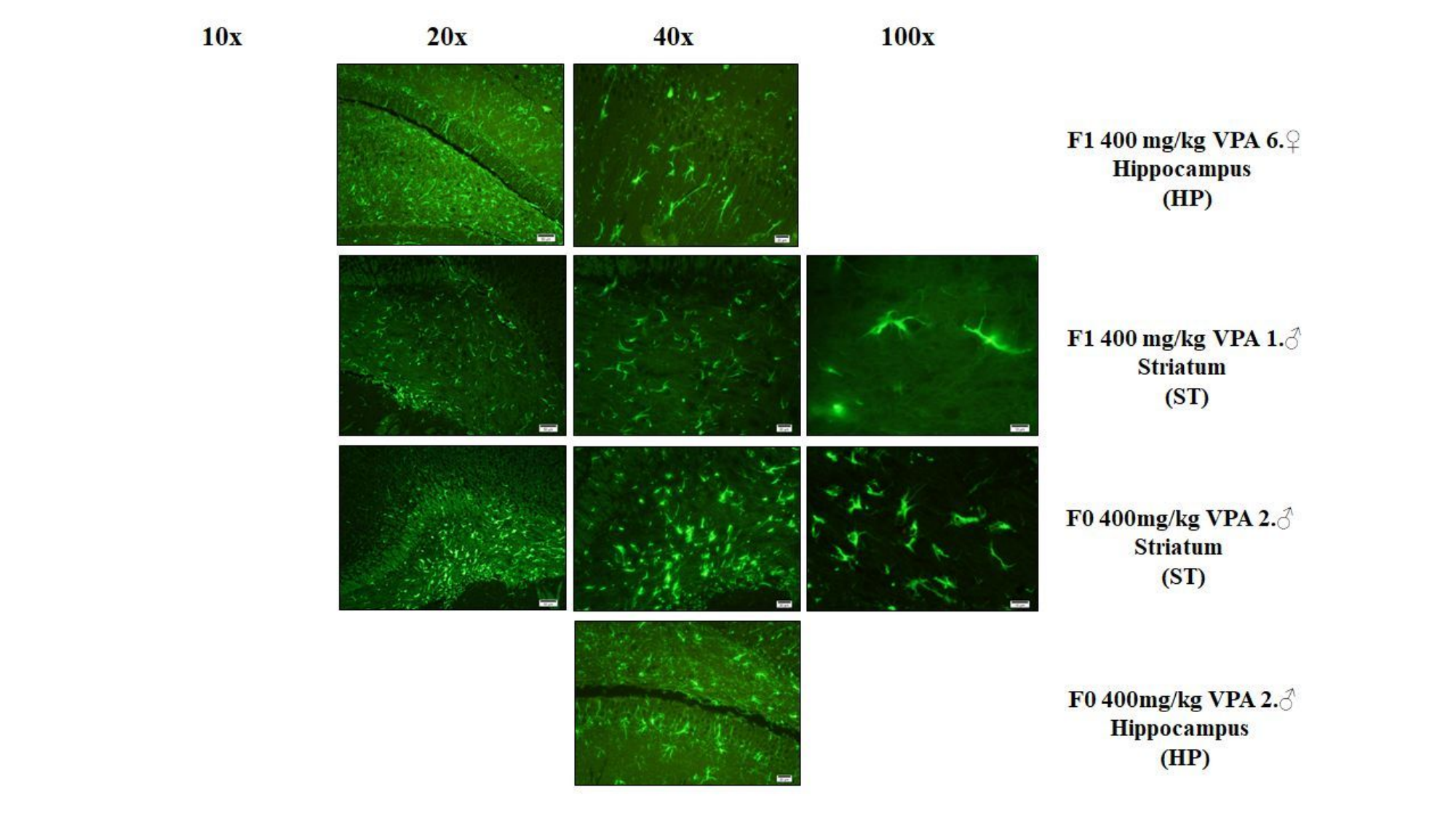

## Slide 15
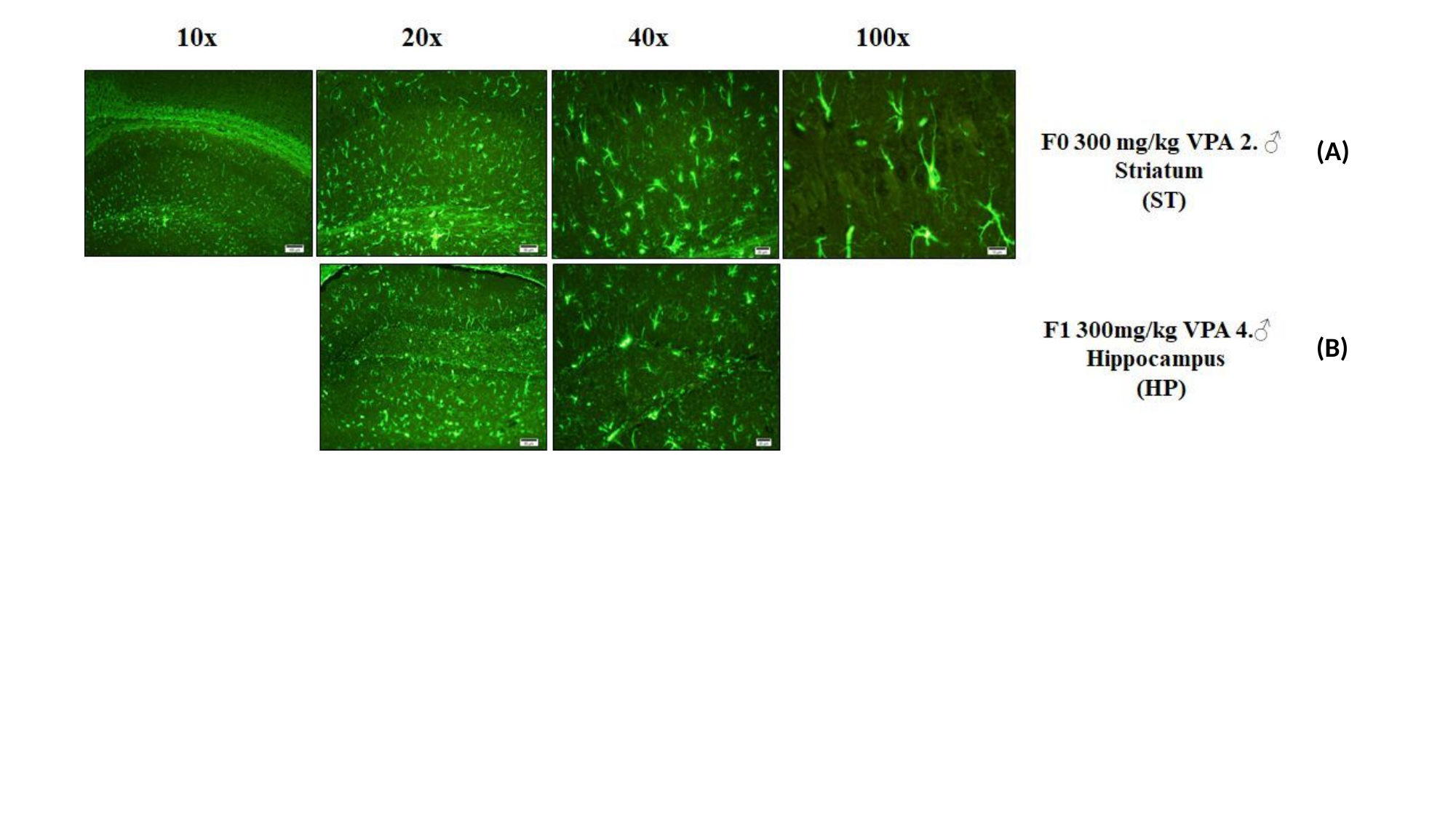

(A)
(B)
